# Defining the filarial N-glycoproteome by glycosite mapping in the human parasitic nematode *Brugia malayi*

**DOI:** 10.1038/s41598-023-34936-9

**Published:** 2023-05-16

**Authors:** Fana B. Mersha, Colleen M. McClung, Minyong Chen, Cristian I. Ruse, Jeremy M. Foster

**Affiliations:** New England Biolabs, Ipswich, MA 01938 USA

**Keywords:** Glycobiology, Parasite biology

## Abstract

N-linked glycosylation is a critical post translational modification of eukaryotic proteins. N-linked glycans are present on surface and secreted filarial proteins that play a role in host parasite interactions. Examples of glycosylated *Brugia malayi* proteins have been previously identified but there has not been a systematic study of the N-linked glycoproteome of this or any other filarial parasite. In this study, we applied an enhanced N-glyco FASP protocol using an engineered carbohydrate-binding protein, Fbs1, to enrich N-glycosylated peptides for analysis by LC-MS/MS. We then mapped the N-glycosites on proteins from three host stages of the parasite: adult female, adult male and microfilariae. Fbs1 enrichment of N-glycosylated peptides enhanced the identification of N-glycosites. Our data identified 582 N-linked glycoproteins with 1273 N-glycosites. Gene ontology and cell localization prediction of the identified N-glycoproteins indicated that they were mostly membrane and extracellular proteins. Comparing results from adult female worms, adult male worms, and microfilariae, we find variability in N-glycosylation at the protein level as well as at the individual N-glycosite level. These variations are highlighted in cuticle N-glycoproteins and adult worm restricted N-glycoproteins as examples of proteins at the host parasite interface that are well positioned as potential therapeutic targets or biomarkers.

## Introduction

*Brugia malayi* is one of three human filarial parasites that cause lymphatic filariasis, a debilitating and chronic neglected tropical disease that currently threatens more than 859 million people in 50 countries worldwide^1,2^. Symptoms in individuals can range from mild to severe including damage to the lymphatic system and kidneys, and eventual clogging of lymphatic vessels with swelling of genitalia and limbs^1,2^. Adult worms residing in the lymphatic system of immunocompetent individuals can survive for an average of 6–8 years. Female worms release millions of microfilariae (immature larvae), which make their way to the bloodstream where they can be ingested by feeding mosquitoes, the insect vector for these parasites. Within mosquitoes, the microfilariae molt and develop through several stages into the infective third larval stage (L3) which can then be transmitted upon a blood meal to other humans. These L3 larvae then molt and mature as they migrate to the lymphatic system where they complete the lifecycle^1,2^. More than 7 billion treatments have been delivered in 66 countries to fight lymphatic filariasis as part of the WHO effort to eliminate the disease^1,3^. Current treatments, while effective at reducing transmission by lowering or eliminating microfilariae circulating in the blood, do not kill the adult worms responsible for the symptoms associated with filariasis^3,4^. Additionally, reports of emerging resistance to current treatments have been made^5,6^. To expand available control measures, additional studies to increase available drug options and drug targets are needed.

Parasite mediated interactions with the mammalian host such as immunomodulation and immune evasion allow *B. malayi* and other filarial parasites to exist for many years in immunocompetent hosts and represent an active area of investigation^7^. Glycoconjugates present at the worm surface, secreted, excreted, or present in extracellular vesicles released by the parasite into the host are all part of crucial mechanisms that allow these parasites to exist for years without clearance^8–11^. Detailed characterization of these critical molecules and their synthesis pathways can highlight candidate biomarkers, therapeutic targets, vaccine molecules or diagnostic tools for further development. For example, the current diagnostic test for lymphatic filariasis is detection of *Wuchereria bancrofti* circulating filarial antigen by a monoclonal antibody^12^. Recent studies showed the epitope recognized is a glycan and additional studies are needed to completely characterize the structure^13^.

An important class of glycoconjugates are N-glycosylated proteins or N-linked glycoproteins. N-glycosylation is a complex post translational modification where glycans are attached to the nitrogen of specific asparagine residues in a protein and play a role in many biological processes including folding, stability and function of the protein^14^. Examples of the roles N-glycosylation plays in host pathogen interactions are well established in viruses and include shielding to allow immune evasion, increasing infectivity, and changing virulence^15^. Particularly, surface N-glycoproteins shown to shield the virus from the host immune system include influenza hemagglutinin glycoprotein^16^, HIV-1 envelope spike protein (gp120/gp41)^17^, and the SARS-CoV2 spike protein^18^. Similar strategies may play a role in metazoan parasites. In addition, the array and modularity of N-glycans confers a level of diversity that can be exploited by the parasite for both short term or long term heterogeneity as was recently demonstrated for *Acanthocheilonema viteae* ES-62^19^ and is likely important for host parasite interactions^14,20^. These adaptable, post translationally modified proteins of the N-glycoproteome are thought to evolve more than the proteome of the organism and have less conservation with other species^21^. Thus, the filarial parasite N-glycoproteome is expected to include surface N-glycoproteins specific to the parasitic lifestyle and critical to interactions with the mammalian host.

For parasitic nematodes, the host parasite interface can be defined as the cuticle or outer exoskeleton covering of the worm, excretory-secretory (ES) components, and extracellular vesicles. Early studies of filarial worm cuticle proteins identified two *B. malayi* glycoproteins. gp29 (Bm2151) is a major surface glycoprotein found in the cuticle of adult worms with homology to the glutathione peroxidases that are part of the oxidative stress response^22^. gp15/400 (Bm6083 or Bm6084, Bma-npa-1) is a nematode polyprotein allergen related glycoprotein found in the cuticle of the adult worms and is thought to play a role in acquiring the required fatty acids that the worms cannot synthesize de novo^22,23^. Recently, gp15/400 was described as an immunodominant antigen targeted by human IgE monoclonal antibodies produced from parasite infected human sera^24^. Later studies broadened the number of cuticle associated proteins but little is known about their glycosylation status^25^. ES-62 (Bm9816) is a major secreted glycoprotein in filarial nematodes^26,27^ and of special interest due to immunomodulatory phosphorylcholine substitution of its N-glycans^8,9,26^. Larger proteomic studies of excreted and secreted proteins^28,29^, extracellular vesicle proteins^30^ and membrane or surface enriched proteins^31^ highlighted more proteins that are exposed to the mammalian host. However, the possibility of glycan-mediated interactions with the host were not addressed in these strictly proteomic analyses. N-linked glycosite mapping in *Caenorhabditis elegans*, a soil living nematode, identified 829 N-linked glycoproteins in one study and 1010 N-linked glycoproteins in a second^21,32^. These studies that identified over a thousand N-glycoproteins included all stages (egg, L1, L2, L3, L4, and adult worms) of the nematode life cycle^33^. A recent glycosite mapping of a Clade V^34^ parasitic nematode, *Haemonchus contortus,* identified 291 N-linked glycoproteins^35^ from only a mixed adult worm sample.

Here, we utilize and expand on these findings by identifying and mapping the *B. malayi* N-glycosites of proteins from total lysates prepared from adult female worms, adult male worms, and microfilariae to characterize the N-glycoproteome and further explore the proteins at the interface of filarial host parasite interactions. Significantly, we show that our data includes known examples of cuticle and immunomodulatory host parasite interacting proteins. We also highlight two different groups of N-glycoproteins. The first set is a group of N-glycoproteins that have ten or more N-glycosites. We used this set to explore N-glycosite occupancy variation among the three studied sample types. The second set is a group of N-glycoproteins that are unique to adult female and adult male worms. We used this set to explore filarial and nematode specific proteins.

## Materials and methods

### *B. malayi* total lysate prep

Approximately 200 female worms, 100 male worms, or 2 million microfilariae (TRS Labs Inc., Athens GA) were resuspended in lysis buffer (100 mM Tris HCl pH 7.5, 100 mM DTT, 4% SDS (v/v)) at 10 ml per 0.3 g wet worm pellet. To begin lysis, samples were frozen on dry ice for 10 min and then thawed at 37 °C four times prior to homogenization with a glass Dounce homogenizer. The homogenized samples were then heated to 100 °C for 5 min and then chilled on ice. Cellular debris and unbroken cells were pelleted at 15,000 × g for 10 min. The protein concentration of each supernatant was determined using Pierce™ 660 nm Protein Assay Kit (ThermoFisher Scientific, Waltham MA).

### Filter aided sample prep (FASP)

Tryptic peptides were produced as described previously^36^. In brief, a maximum 400 µg of each protein lysate was buffer exchanged into urea buffer (100 mM Tris HCl, pH 8.2, 8 M Urea.) using Microcon 30 K centrifugal filters (Millipore Sigma, Burlington, MA). This was followed by alkylation of cysteine resides with 50 mM iodoacetamide (Sigma-Aldrich, St. Louis, MO) in the same urea buffer in the dark for 20 min. Excess iodoacetamide was washed away and then buffer was again exchanged, this time into 50 mM ammonium bicarbonate. The protein mixture was then digested with Trypsin (P8101S, New England Biolabs, Ipswich, MA) in a 1:100 ratio of enzyme to protein at 37 °C overnight and the resulting peptides collected. Peptide concentration was determined by Pierce Colorimetric Peptide Assay quantitation kit. (ThermoFisher Scientific).

### Fbs1-mediated N-glycopeptide enrichment (N-FASP)

N-glycopeptides were enriched as described previously^37^. In brief, 100 µg of total lysate peptide mix was mixed with 200 µg of Fbs1 GYR in a Microcon 30 K centrifugal filter and incubated at 4 °C for 2 h. The unbound peptides were removed by centrifugation. After washing to ensure all unbound peptides were removed, the enriched N-glycosylated peptides were eluted with 50% formic acid. The elution was then lyophilized to remove the formic acid and water.

### N-glycan release by PNGase F in the presence of ^18^O water

Peptides from 100 µg of lyophilized total peptide mix (Total) or the Fbs1 GYR enriched peptide mix (Fbs1) from 100 µg starting total peptide mix were resuspended in 100 mM ammonium bicarbonate prepared with ^18^O water (Cambridge Isotope Laboratories, Inc. Andover, MA). 1000 units of PNGase F (P0704, New England Biolabs) buffer exchanged into 100 mM ammonium bicarbonate prepared with ^18^O water were added to a 50 µl final reaction that contained either Total or Fbs1 peptide mix. The reactions were incubated at 37 °C for 1 h. Samples were run on LC-MS/MS as noted below immediately to avoid spurious chemical deamidation events.

### LC-MS/MS

Peptides from each of the six samples were analyzed by mass spectrometry. For each sample, 6% of the resultant peptide volume was loaded onto a reversed phase analytical column (Ion Opticks Aurora UHPLC column, 25 cm × 75 µm ID, 1.6 µm C18) via a Proxeon Easy-nLC 1000 (Thermo Scientific). The column was housed in a Sonation Column Oven (Sonation Lab Solutions) and kept at 50 °C. Peptides were eluted over a three-hour window consisting of a 156-min linear gradient from 2 to 35% B, a three-minute gradient to 85% B and an isocratic flow at 85% B for seven minutes where mobile phase A was water containing 0.1% formic acid and mobile phase B was acetonitrile containing 0.1% formic acid. The eluted peptides were introduced into a Q Exactive mass spectrometer (Thermo Scientific) by electrospray using a Nanospray Flex ion source (Thermo Scientific) at a flow rate of 400 nL/min. The ten most abundant ions from each full scan (70 k resolution, scan range 400–1600 m/z) were selected for fragmentation by HCD (higher energy collisional dissociation), and fragmentation spectra were acquired with 35 k resolution. A stepped normalized collision energy of 20, 30, and 40 was used. Charge states of one and greater than eight were excluded. Dynamic exclusion was set to 30 s. Each sample was analyzed with three technical replicates and the spectra from each triplicate set were combined for analysis.

### Data analysis

Spectral data were searched against the combined *B. malayi* proteome (brugia_malayi.PRJNA10729.WBPS10.protein.fa) from Wormbase^38^ and the *Wolbachia* endosymbiont of *B. malayi* proteome from NCBI^39^, both downloaded in December 2019 and then analyzed using Byonic software (Protein Metrics, Cupertino, CA). Common contaminants such as keratins, caseins, trypsin, and BSA were removed from the analysis. Protein output was set at 1% FDR. Other parameters used for each mass spectrometry dataset were Cleavage site = RK, C-terminal side = semi specific; missed cleavage = 2; Mass tolerance = 10 ppm; QTOF/HCD fragmentation with 0.02 Da fragment mass tolerance. Fixed modifications = carbamidomethyl @ C/ + 57.021464. Variable Modifications = deamidated:^18^O(1) / + 2.988261 @ N, oxidation/ + 15.994915 @ M, deamidated / + 0.984016 @ N and Q, acetyl @Protein N-term / + 42.010565, Gln- > pyro-Glu/ -17.026549, amidated @ D and E / -0.984016. The mass spectrometry proteomics data have been deposited to the ProteomeXchange Consortium via the PRIDE^40^ partner repository with the dataset identifier PXD039002 and 10.6019/PXD039002.

We filtered the results such that peptides were required to have a Byonic score > 200 and a peptide length > 4 for the Fbs1 enriched samples. For the Total samples, which had a higher complexity, we used a Byonic score of > 300 and a peptide length > 4. The Byonic score is a gauge of the accuracy of the peptide spectrum match^41^. Single unique peptides that were associated to only one protein, in one replicate of the sample, and were not present in the remaining five samples are marked by an asterisk in Supplemental Table S1 and not included in further data analysis. To compare the proteins identified by LC-MS/MS to previously published proteomic studies^28–31,42,43^ that used different protein IDs, we cross referenced the Wormbase^38^ and UniProt^44^ databases to correlate Wormbase ID from our data to the PUB_loci/pub_locus numbers or to UniProtKB ID for the *B. malayi* proteome and to the UniProt database to correlate NCBI ID with UniProtKB ID or wBm gene numbers for the *Wolbachia* proteome. The synonyms we found associated with each protein in our dataset used for cross referencing are listed in Supplemental Table S2.

We used NetNGlyc-1.0^46^ to find the canonical N-X-S/T glycosites present in each N-glycoprotein. We used Weblogo^47^ to create a sequence logo for extracted -10 to + 10 amino acid sequences. We used BioVenn^48^ to generate a proportional Venn diagram. We used TMHMM-2.0^49^ to predict transmembrane domains. We used UpsetR^50^ to generate UpsetR intersecting set plots. The gene ontology identification and functional enrichment analysis was performed using g:Profiler (version e105_eg52_p16_e84549f.) with g:SCS multiple testing correction method applying significance threshold of 0.05^51^. We used ggplot^52^ to generate scatter plots from gene enrichment data analysis. We used DeepLoc to predict subcellular localization for each N-glycoprotein^53^. We used Parasite Biomart at Wormbase to search for both *C. elegans* and *H. contortus* orthologs to identified *B. malayi* N-glycoproteins^54^. We used Clustal Omega to generate a multiple sequence alignment^55^.

## Results and discussion

### Fbs1 enrichment

The improved N-glyco FASP method uses an Fbs1-GYR mutant that specifically binds N-glycosylated peptides. This engineered protein is selective for a broad range of N-linked glycopeptides and was shown to have 2.2 fold higher enrichment and less bias compared to the standard N-glyco FASP protocol that uses a mix of lectins ConA, WGA and RCA_120_^37^. It was also shown that the Fbs1 enrichment method is more suitable for N-glycosite analysis than the less selective methods (HILIC or hydrazide) that allow enrichment of O-linked glycans or other hydrophilic molecules^37^.

We analyzed samples treated with PNGase F in the presence of ^18^O water from both unenriched total proteome samples (Total) and post Fbs1 enrichment samples (Fbs1) by LC-MS/MS for adult female worms, adult male worms, and microfilariae. PNGase F is a peptide N-glycosidase that specifically releases oligomannose, hybrid glycans, and complex glycans attached to an asparagine by cleaving at the amide bond^56^. The cleavage of the glycan with PNGase F in ^18^O water generates a deamidation event as the asparagine is converted to aspartic acid with the incorporation of ^18^O. This results in + 2.98 Dalton addition on the primary amino acid sequence of the peptide^57^ readily detected by mass spectrometry. Peptides containing an asparagine with this + 2.98 Dalton addition are hereafter called N + 3 peptides. Without ^18^O water, the PNGase F cleavage results in + 0.98 Dalton deamidation which can also be a result of unwanted spontaneous deamidation of asparagine during sample processing. Though PNGase F is unable to release N-glycans when the α (alpha) 1–3 fucose is present on the core N-acetyl glucosamine (GlcNAc) of an N-glycan, we did not use a second enzyme like PNGase A because α (alpha) 1-3 fucosylation of the core GlcNAc is absent in *B. malayi*^58^ and other filarial nematodes^59^.

**Figure 1:**
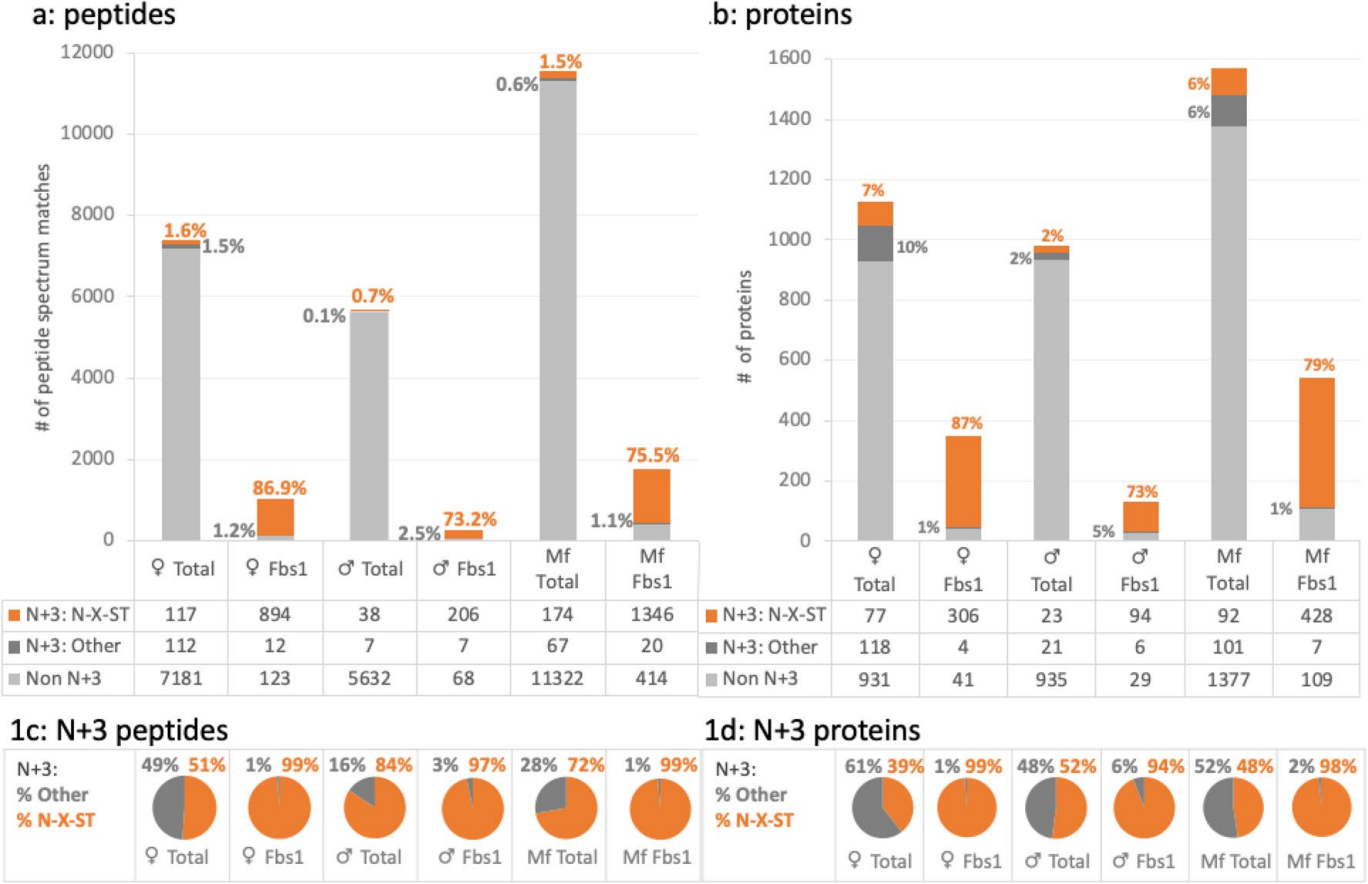
Fbs1 enrichment. Each bar represents the mean of peptide spectrum matches in (**a**) or mean of identified proteins in (**b**) of both Total (no enrichment) or Fbs1 enriched samples for female worms, ♀, male worms, ♂, and microfilariae, Mf. In light gray, labeled Non N + 3, are the peptides or proteins without the N + 3 modification. In dark gray, labeled N + 3: Other, are the noncanonical N + 3 containing peptides or proteins without an N-X-S/T glycosite. In orange labeled N + 3: N-X-S/T are the N-glycosite peptides or proteins with both an N + 3 modification and an associated canonical N-X-S/T glycosite. The percentage in dark gray next to the bar represents the N + 3: Other percentage in the whole sample. The percentage in orange next to the bar represents the N + 3: N-X-S/T peptides or proteins in the whole sample. The pie charts represent the percentage of Other vs. N-X-S/T for the N + 3 peptides in (**c**) or N + 3 peptide containing proteins in (**d**).

The utility of the Fbs1 enrichment is illustrated in Fig. 1a where the number and percentage of peptide spectrum matches with and without an N + 3 modification in each sample is summarized. The complete data can be found in Supplemental Table S1. As detailed in the “Materials and methods” section, to ensure we could compare the samples, we normalized the inputs by determining peptide concentration for all samples prior to enrichment. Fbs1 enrichment improves both the overall number of N-glycosylated peptides and the quality of the data. Without enrichment, the number of N + 3 peptides in the Total samples is limited to 1% to 3% of all peptides. Even with tens of thousands of peptides identified in the Total samples, the final number of N + 3 peptides identified falls between 45 and 241 with the least present in the male worm sample. The Fbs1 enriched samples, in contrast, have 213 to 1366 N + 3 peptides representing a four to five fold increase in available N + 3 peptide data when compared to the Total samples. In addition, to define the N + 3 peptides as N-glycosites, we required a canonical N-glycosylation site motif, N-X-S/T, where X could be any amino acid other than proline^21^. (The N-X-S/T sequence will hereafter be referred to as a canonical N-glycosite and an N + 3 peptide with a canonical N-glycosylation site will be referred to as an identified N-glycosite. Specific identified N-glycosites will be listed with the position of the asparagine in the amino acid sequence followed by its N-X-S/T sequence. e.g., 67 NET) Thus, to be an identified N-glycosite, all N + 3 peptides were assessed for including a canonical N-glycosite and are noted in Fig. 1a in orange. These results exemplify the power and utility of the Fbs1 enrichment method in identifying N-glycosites as there is high concordance of the N + 3 modification with a canonical N-glycosite in the Fbs1 enriched samples. Across the three Fbs1 samples, 74–87% of all peptides in the sample have an N + 3 modification present on a canonical N-glycosite. Figure 1c focuses on peptides with N + 3 modification and shows that the percentage of N + 3 modified peptides with a canonical N-glycosite in Fbs1 samples is 98–99%. In the Total samples with a higher sample complexity, the concordance is substantially lower. Expectedly, only 0.7% to 1.6% of all peptides in these unenriched samples have an N + 3 modification present on a canonical N-glycosite (Fig. 1a). However, even when only peptides with an N + 3 modification are analyzed, only 51–84% of the N + 3 modified peptides in the Total samples are found with a canonical N-glycosite (Fig. 1c). This greater background is despite a higher Byonic score threshold for the peptides from the Total samples being utilized to increase stringency. Consequently, the enrichment of peptides by Fbs1 both increases the number of identified N-glycosites and decreases the background.

The Fbs1 enrichment results are mirrored when looking at the results at the protein level. Proteins identified by a single unique peptide in only one replicate of the six samples were excluded from our analysis (Supplemental Table S1, * Single peptides). In the Fbs1 enriched samples, a higher percentage of proteins had an N + 3 modification and ranged from 78 to 88% as seen in Fig. 1b. In contrast, in the Total samples, the N + 3 modification was only found in 4% of the male worm proteins and 13–18% of the female worm and microfilarial proteins. For the Total samples, Fig. 1d shows that there were relatively similar numbers of N + 3 peptide-containing proteins without a canonical N-glycosite as there were with an N-X-S/T sequence. With Fbs1 enrichment, only 1–6% of proteins had N + 3 peptides without a canonical N-glycosite. This results in four to five-fold more N-glycosylated proteins identified in Fbs1 enriched samples versus Total or unenriched samples with equivalent input amounts analyzed.

### Identification of N-glycosites and N-glycoproteins

In all, over 2000 different proteins were identified in the six samples analyzed (Supplemental Table S2). After extracting all proteins with an N + 3 modification and requiring a canonical N-glycosite, we identified 582 N-glycosylated proteins with 1273 N-linked glycosites (Supplemental Table S3). It should be noted that this data was generated from technical replicates of a pooled sample from female worms, male worms, and microfilariae. In comparison, with over 1000 N-glycoproteins in the *C. elegans* N-glycoproteomes^21,32^ that encompass all life stages and the *H. contortus* N-glycoproteome with 282 N-glycoproteins^35^ which represent adult worm samples, we believe this *B. malayi* N-glycoproteome from adult worm and microfilarial samples provides a good snapshot of the filarial N-glycoproteome present at the mammalian host stage.

As is common with other studied N-glycoproteomes, the distribution of N-glycosites favored NXT with 791 sites (62%) as compared to NXS with 482 sites (38%)^21^. We extracted the sequences 10 amino acids upstream and downstream of the identified N-glycosites to search for additional conserved motifs^47^ (Supplemental Table S3, Supplemental Fig. S1). The data showed the expected N-X-S/T where X could be any amino acid but proline and no additional motifs. Proteins from the *Wolbachia* endosymbiont were found in the samples but as expected we did not identify any of these as N-glycoproteins (Supplemental Table S2).

**Figure 2:**
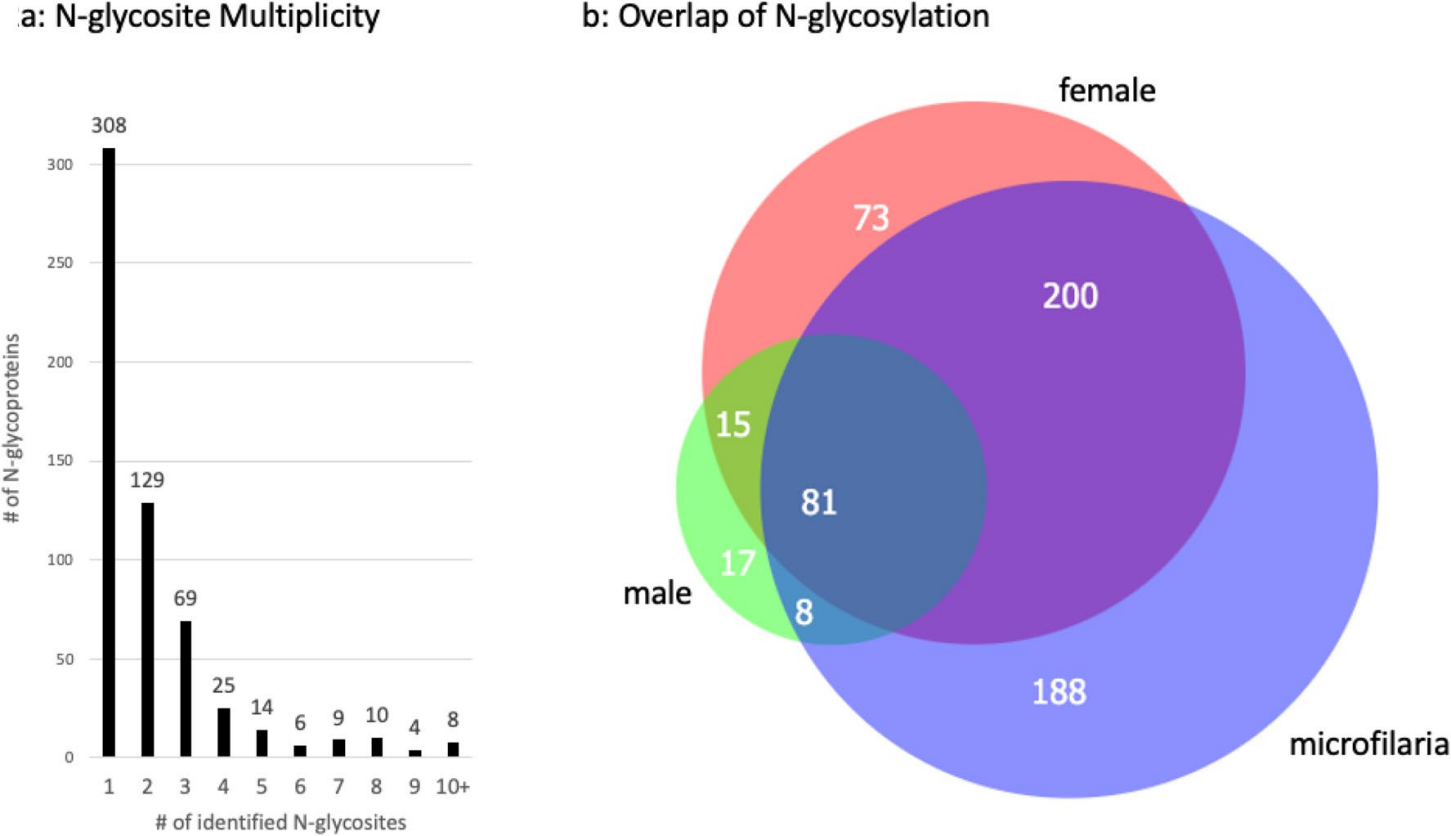
N-glycoproteins (**a**) The distribution of N-glycoproteins that have 1 through 10 + N-glycosites. (**b**) Area proportional Venn diagram overlap comparison of the 582 identified N-glycoproteins from standardized amounts of *B*.* malayi* female worms, male worms, and microfilariae analyzed in this study.

53% of the N-glycoproteins have one N-linked glycosite while the remainder have two or more N-linked glycosites (Fig. 2a). Eight proteins had ten or more N-linked glycosites and are listed in Table 1. These highly glycosylated proteins demonstrate the increased number of N-glycosites that can be discovered using Fbs1 enrichment. For all eight proteins, there are more N-glycosites identified in the Fbs1 samples than in the Total samples. Though the increased number of N-glycosites revealed by Fbs1 enrichment is readily apparent in highly glycosylated proteins, this trend remains the same for most N-glycoproteins. In fact, only 33 or 2.6% of the 1273 N-glycosites were exclusively found in Total samples but not in Fbs1 samples. These few proteins are noted with an asterisk in the sample columns of Supplemental Table S3.

**Table 1:** *B. malayi* N-glycoproteins with ten or more N-glycosites.

Wormbase ID	Description	GO terms	aa length	Canonical	# of identified N-glycosites							
					All	Female		Male		Mf		*C. elegans* ortholog
						Total	Fbs1	Total	Fbs1	Total	Fbs1	
Bm10822b	ortholog of *C. elegans* dig-1, K07E12.1; Ig-like, Fibronectin type-III, VWFA domains	MF: calcium ion binding and protein binding CC: membrane	6469	32	21	0	16	1	4	7	18	10
Bm2376a	ortholog of *C. elegans* igdb-1 and igdb-2; Ig-like, Fibronectin type-III domains	MF: protein binding CC: membrane	1477	24	17	1	13	0	2	4	17	11
Bm4801a	ortholog of *C. elegans* let-805, H19M22; Fibronectin type-III domains	MF: protein binding CC: membrane	4470	25	15	3	10	2	5	10	14	14
Bm3694	ortholog of *C. elegans* lrp-1, F29D11; LDL-receptor class B, EGF-like domains	MF: calcium ion binding and protein binding CC: membrane	4762	34	14	0	5	0	8	0	10	16
Bm5908	ortholog of *C. elegans* lam-3, T22A3; laminin EGF like, laminin IV type A domains	BP: Cell adhesion	3332	28	13	1	8	0	1	2	12	16
Bm6131	ortholog of *C. elegans* clec-78, F47C12; C-type lectin, CUB, Sushi, EGF-like domains	MF: calcium ion binding and protein binding CC: membrane	3579	37	13	0	6	0	0	0	13	4
Bm7191	ortholog of *C. elegans* Y92H12BR.3; transmembrane domain	CC: membrane	641	22	12	2	10	0	1	4	10	9
Bm9007	ortholog of *C. elegans* mup-4; EGF-like, VFWA, SEA domains	MF: Lipid transporter	1940	21	10	0	7	0	2	3	10	2
		BP: Biological Process										
		MF: Molecular Function										
		CC: Cellular Component										

### N-glycosite occupancy

Another notable feature in Table 1 is the variability in N-glycosite occupancy across samples. The total identified N-glycosites across all six samples is listed in the 6th column labeled All. Comparing this total to the N-glycosites found in the individual samples shows the variation in N-glycosites between these samples. For two proteins, Bm2376a and Bm6131, the total identified N-glycosites matches the total found in microfilariae. A smaller subset of these N-glycosites, though, are found in adult male and adult female worms. For the other six proteins, there is a mixture of individual and overlapping N-glycosites that sum to the total number across all samples.

**Figure 3:**
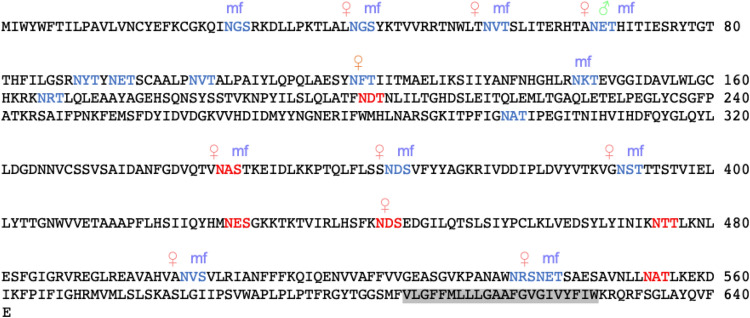
N-glycosite occupancy of Bm7191: The full protein sequence of Bm7191 is shown with the predicted transmembrane region highlighted in gray^49^. N-X-S/T sites are colored blue in protein sequence if predicted as an N-glycosite and colored in red if threshold determined by jury agreement using nine neural networks and scoring higher than a set threshold by NetNGlyc-1.0^46^ was not met. N-glycosites found in female worms (red coloured ♀), male worms (green coloured ♂) and microfilariae (blue coloured mf) are indicated above the sequence.

Figure 3 illustrates the variability in N-glycosite occupancy for Bm7191. This protein is predicted to be a membrane protein with the first 604 amino acids of the protein oriented outside the membrane and a single transmembrane region from 605 to 627 highlighted in gray^60^. It has 22 canonical N-glycosites all found within the region predicted to be outside the membrane^46^. While a total of 12 N-glycosites were identified, mapping on the protein sequence shows that only one site at 67 NET is occupied in female worms, male worms, and microfilariae. In microfilariae, two N-glycosites at 26 NGS and 145 NKT are uniquely occupied and in female worms, two other N-glycosites at 120 NFT and 443 NDS are uniquely occupied. The remaining seven sites are occupied in both female worms and microfilariae. Two sites at 345 NAS and 443 NDS were below the set threshold for N-glycosite prediction but were identified as N-glycosites showing that the threshold for N-glycosite prediction in some cases may need to be lowered. The variability in N-glycosite occupancy needs to be explored further to determine its biological significance.

### N-glycosylation variability

The overlap of proteins observed to be N-glycosylated in male, female, and microfilariae is shown in Fig. 2b. While 81 or 14% of the proteins are found N-glycosylated in all three samples, almost half of the proteins (32% in microfilariae, 12% in female and 3% in male) are only N-glycosylated in one of the three samples. The greatest overlap of 34% of all N-glycoproteins is between the female worm and microfilarial samples. This overlap is likely confounded by the intrauterine microfilariae that are present in female worms and should be kept in mind when specific N-glycosites are found in common between female and microfilariae in individual N-glycoproteins. Of the three sample types studied here, microfilariae have the largest number of N-glycoproteins at 477 and the most unique N-glycoproteins at 188. This could be because microfilariae need to evade the immune system while migrating from the lymphatics to the peripheral circulation and subsequently develop further within a mosquito vector. Male worms have the least N-glycoproteins as well as the least N-glycosites (Table 1, Fig. 1a,c). It is not clear what is responsible for this observed lower level of N-glycosylation. Nevertheless, there are 17 male worm N-glycoproteins that are not found N-glycosylated in either the Total or Fbs1 samples from adult female worms or microfilariae.

### Previous proteomic data set comparisons, gene ontology, and cell localization

**Figure 4:**
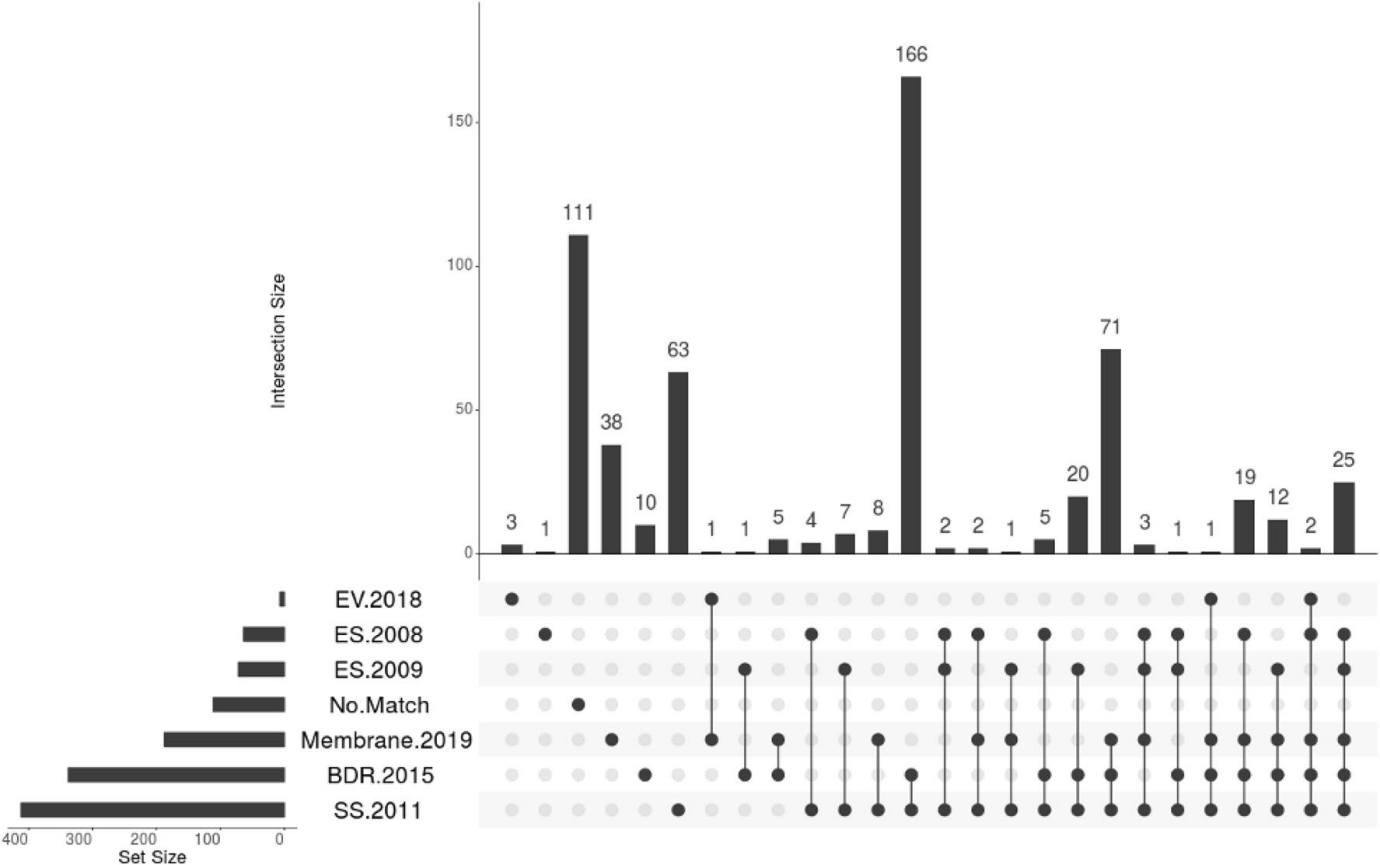
UpSetR data analysis of identified *B. malayi *N-glycoproteins with previously published *B. malayi* proteomes: EV 2018^30^ is the extracellular vesicle proteome**.** ES 2008^29^ and ES 2009^28^ are the excretory and secretory proteome sets. Membrane. 2019^31^ is the surface and membrane proteome sets; BDR 2015^42^ is the body wall, the digestive tract and the reproductive tract proteome sets. SS 2011^43^ is the stage specific proteome sets. No Match indicates identified N-glycoproteins not found in these six proteomic studies. The set size shown on the left indicates the number of proteins in each set. The bars that are above single dots show the number of N-glycosylated proteins unique to that proteome. The bars that are above multiple joined dots show the number of N-glycosylated proteins in common with those proteomes^50^.

Comparing the 582 N-linked glycosylated proteins to previously published *B. malayi* proteomes, we found 111 proteins (19%) had not been previously identified in any proteomic study (Fig. 4, No Match). This shows the power of Fbs1 enrichment to mine the proteome for low abundance or difficult to find proteins. Our identification of N-glycosylated proteins also adds context to the previously published proteomes; we added glycosylation status to 471 previously reported proteins. For example, 136 N-glycoproteins overlapped with excretory secretory proteomes and 188 overlapped with a membrane proteome. This information can help us understand where these N-glycoproteins are found, and at which stage they are present in the worm.

**Figure 5:**
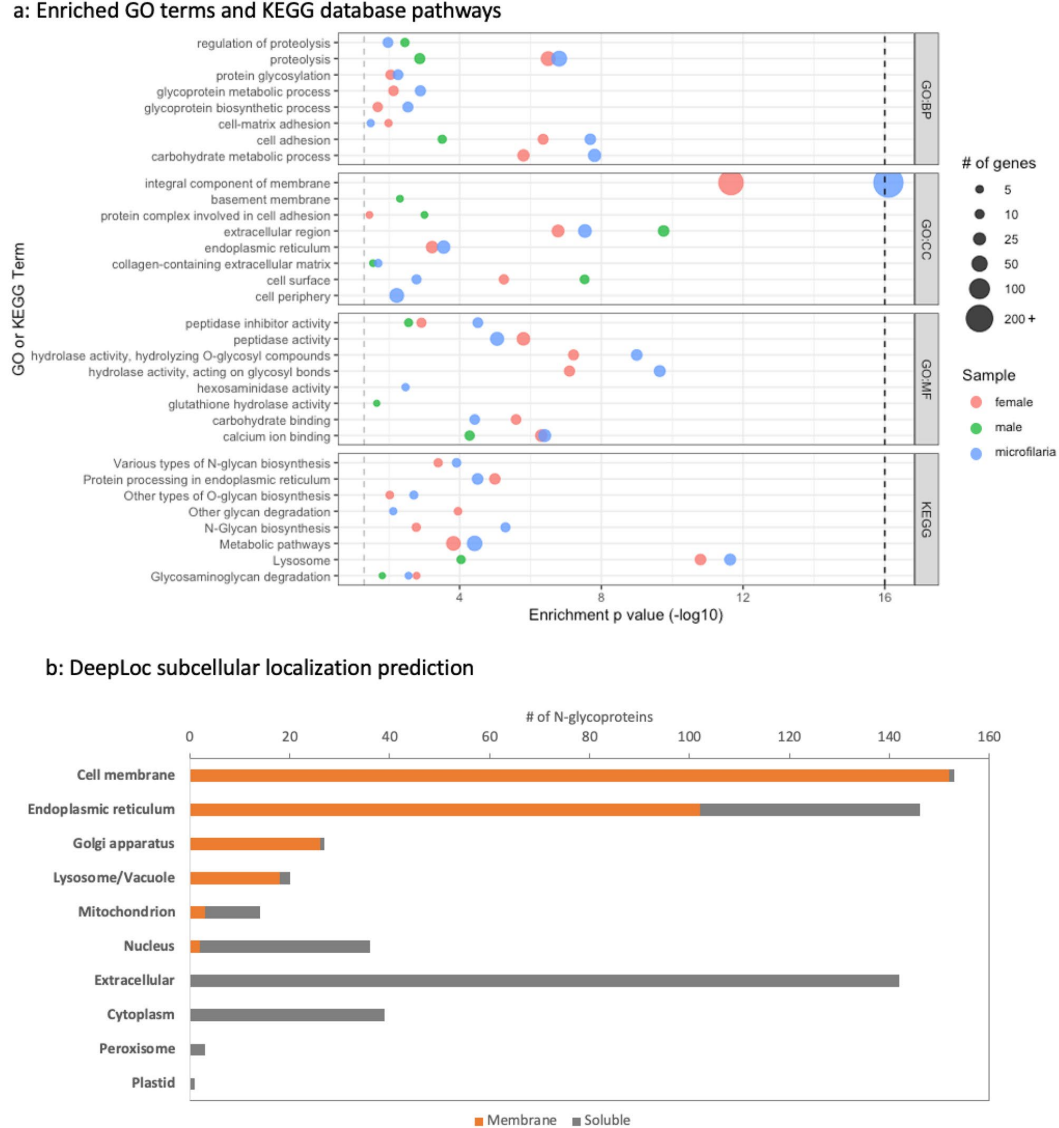
Gene ontology and subcellular localization prediction and analysis (**a**): Scatter plots illustrate enriched GO terms and KEGG database pathways. The vertical axis represents the enriched terms in each category, and the horizontal axis represents the enrichment *p* value. *p* value was capped at 16 as noted by the dark vertical dashed line. The size of dots shows the gene number and the color shows the sample type. GO:BP set are the top Biological Process gene ontology terms, GO:CC set are the top Cell Component gene ontology terms, GO:MF are the top Molecular Function gene ontology terms and the KEGG set are the top KEGG pathways (**b**): Proportional bar graph indicates number of N-glycoproteins in ten different subcellular locations with orange indicating predicted membrane proteins and gray indicating soluble proteins.

To analyze gene enrichment data and explore function and localization information, gene ontology data annotation for the N-glycosylated proteins was obtained and gene enrichment analysis was preformed using g:Profiler^51^ (Supplemental Table S4, Fig. 5a). While absent for about a third or 179 of the N-linked glycoproteins, 403 N-linked glycoproteins had some gene ontology annotation or KEGG pathway^45^ information. This included Cell Component annotation for 329 or 57% of the N-glycoproteins where enrichment analysis shows proteins that are membrane associated, extracellular or cell surface proteins are overrepresented; Biological Process annotation for 148 or 25% of the N-glycoproteins and Molecular Function annotation for 123 or 21% of the N-glycoproteins where proteins involved in proteolysis, glycosylation and adhesion are overrepresented. Similarly, KEGG pathways^45^ important in glycosylation and protein degradation are also overrepresented. As expected for N-glycosylated proteins, this confirms that these proteins have characteristics expected in host parasite interacting proteins.

Furthermore, we used DeepLoc-1.0 which uses a sequence based algorithm to predict subcellular localization^53^. This annotation extended subcellular localization predictions to all 582 proteins (Supplemental Table S5). More than 50% of the proteins are predicted to be membrane proteins and are mainly divided into cell membrane, endoplasmic reticulum, Golgi, and lysosome/vacuole (Fig. 5b). Of the remaining 278 N-glycoproteins that were predicted to be soluble proteins, approximately half are predicted to be extracellular proteins. Gene ontology, subcellular localization predictions and the information from previous proteomic studies helps to gauge N-glycoproteins for their potential host parasite interactions.

### Cuticle proteins

The two cuticle proteins mentioned earlier, gp29, a probable glutathione peroxidase and gp15/400, a nematode polyprotein allergen related protein, are present in all the samples analyzed. Glycosite mapping of gp29 (Bm2151a) indicates variable N-glycosylation; in female worm samples where this protein is abundant (40 unique peptides in the Total sample and 36 unique peptides in Fbs1 sample), both canonical N-glycosites^46^ at 39 NQT and 92 NGT are glycosylated in both Total and Fbs1 samples while in male worms (8 unique peptides in the Total sample and 4 unique peptides in Fbs1 sample), peptides in both the Total and Fbs1 samples show that only site 39 is glycosylated and in microfilariae (4 unique peptides in the Total sample and 2 unique peptides in Fbs1 sample) peptides in the Fbs1 sample show that only site 92 is glycosylated. Since no aglycosylated peptides that overlap site 92 in male Total samples or site 39 in microfilariae Total samples are identified, we can’t conclusively confirm the absence of glycosylation but would expect Fbs1 enrichment to have enriched those peptides if they had been N-glycosylated. As noted earlier, we cannot differentiate in female samples if the N-glycosite is present in female tissue only, in intrauterine microfilaria, or in both. So, in this case, site 92 in the female sample may be due to the intrauterine microfilaria. With 58 unique peptides identified in female Total, 26 unique peptides in male Total, and 17 unique peptides in microfilariae Total, gp15/400 (Bm6084) is a relatively abundant cuticle protein. It is reported to bind lipids and has 16 multiple tandem repeats of a nematode specific domain ABA-1^22,23^ that contains a single canonical N-glycosite within the repeat domain. Our data shows that this NLT glycosite is found both aglycosylated and glycosylated in female and microfilariae samples and glycosylated in male samples. Because of the tandem repeats, though, we cannot determine how many of these 16 repeated N-glycosites are occupied. Additionally, gp15/400 has 8 other canonical N-glycosites^46^ not located in the tandem repeats. Four of these sites at 139 NDS, 359 NGS, 527 NVT and 2867 NHS are glycosylated in female worms, while three sites at positions 139, 359 and 527 are glycosylated in microfilariae and only two at 527 and 2867 are glycosylated in male worms. In contrast, *C. elegans* ortholog, npa-1, with an RNAi extended lifespan phenotype^61^ has tandem repeats but no canonical N-glycosites^46^ opening the possibility that N-glycosylation of gp15/400 has a specialized role in *B. malayi*.

We also checked other cuticle proteins identified by Page et al. as potential cuticle biosynthesis and molting targets^25^ and highlight three additional cuticle N-glycoproteins. First, Bma-PHY-1 (Bm3843) has been shown to be important for cuticle development through RNAi studies in *B. malayi*^62^. It is an ortholog of the *C. elegans* dpy-18 which is a prolyl-4-hydroxylase α-subunit important in collagen biosynthsis^63,64^. *C. elegans* dpy-18 has also been shown to be N-glycosylated although at a different glycosite^21^. Peptides for Bma-PHY-1 were found in female, male, and microfilarial samples, but out of 2 possible canonical N-glycosites, glycosylated peptides were only found for the glycosite at 157 NAS and only in female and microfilarial samples. Second, Bm-MLT-7 (Bm7474) is an ortholog of *C. elegans mlt-7* which has an RNAi early larval lethality phenotype. It is predicted to have heme binding activity and essential peroxidase activity important for cuticle synthesis and molting^65^, and is shown to be N-glycosylated at both canonical N-glycosites present in the protein^21^. In our data, peptides for Bm-MLT-7 were found in female, male, and microfilarial samples and while both canonical N-glycosites at 95 NST and 477 NIS were N-glycosylated in female samples, only 95 was N-glycosylated in microfilariae. Third, Bm-CPZ-1 (Bm3754) is predicted to be a cathepsin based cysteine protease important in ecdysis, cuticle development and post embryonic body morphogenesis with RNAi phenotype in *B. malayi* of reduced release of microfilariae^66^. Its orthologs, *C. elegans cpz-1* and *O. volvulus cpz-1,* have RNAi phenotypes showing molting defects^67,68^. *C. elegans cpz-1* has been shown to be N-glycosylated at one site^21^. In our data, this protein was found in all three data sets and N-glycosylated peptides for the only canonical N-glycosite at 187 NYT were found in female worms and microfilariae. Although there is no confirmation from aglycosylated peptides, the lack of N-glycosylation found in male Fbs1 samples for these three cuticle proteins reinforces both the observed lower glycosylation level in male worms and the variability in N-glycosylation of proteins between male worms, female worms, and microfilariae.

### Immunomodulatory proteins

Previous studies have identified ES-62, Bm-mif-1, Serpin and BmVal1 as important proteins that modulate the host immune system^28^. ES-62 (Bm9816) is a predicted leucyl aminopeptidase with host immunomodulatory activity that has been linked to the phosphorylcholine on its N-glycans^27,28^. Recently, the N-glycosylation state of the *A. viteae* ES-62 from mixed adult sample was characterized and showed both heterogeneity of PC-containing glycans and differences in site occupancy at the four N-glycosylation sites of the protein^19^. While *A. viteae* ES-62 and Bm9816 are 71% identical by BlastP^39^, their N-glycosites do not overlap highlighting that even with proteins with a high level of identity, N-glycosites can also differ. In our data, Bm9816 is found in all three data sets but while relatively abundant in the female sample (20 unique peptides in Total sample) and in the microfilarial sample (17 unique peptides in Total sample), only one unique peptide was identified in the male sample. N-glycosylated peptides for 4 N-glycosites at 30 NDT, 128 NIT,146 NVS, 241 NHT were found for ES-62 in the female and microfilarial samples out of a possible 6 canonical N-glycosites present in the protein. The N-glycosite at 241 NHT is one of the few instances that an N-glycosite was present in both the female Total and microfilaria Total samples and not in either Fbs1 sample. No glycosylated peptides were identified in the male sample. Bm-mif-1 (Bm6870) is a filarial secreted macrophage migration inhibitory factor and was found to be a major antigen of parasitic worm infection^24,69^. This protein is relatively abundant in Total samples (19, 8, 22 unique peptides in female, male and microfilarial, respectively) but was not found in Fbs1 enriched samples indicating that at least in adult worms and microfilariae, Bm-mif-1 is not N-glycosylated even though it has two canonical N-glycosites. It may be that it is N-glycosylated at a different life stage or under conditions not studied here. Serpin (Bm9380) is a microfilarial serine protease inhibitor thought to act on enzymes of human neutrophils^70^. As expected, peptides from Serpin were not found in the female or male data sets but were present in both microfilarial Total and Fbs1 enriched samples with N-glycosylation identified at both canonical N-glycosites, 21 NST and 266 NSS. BmVal1 (Bm4233b) has been shown to be an important protein in host parasite interactions with a high host antibody response^71^. Also, in a recent *B. malayi* spatial transcriptomic study, BmVal1 was identified amongst female head-enriched gene transcripts important for feeding, sensory, secretory, and reproductive behaviors and is therefore a promising adult worm druggable target^72^. BmVal1 glycoprotein has 2 canonical N-glycosites and has been produced recombinantly in plant cells with both sites glycosylated and its protein structure has been determined^71^. In our study, both N-glycosites at 52 NGT and 138 NLT were glycosylated in the female and microfilarial samples while in male samples, there was only data to confirm that the second site at 138 is glycosylated.

Bm5654 is an ortholog of an aminopeptidase (antigen H11) shown to be an important part of an immunoprotective extract in *H. contortus*^73,74^*.* This protection has been linked to N-glycosylation^74^. Bm5654 is found in all three data sets and is abundant in the female sample (43 unique peptides in Total sample) and in the male sample (12 unique peptides in Total sample), but only one unique peptide was identified in the microfilarial total sample. N-glycosylated peptides for 8 N-glycosites were found in the female samples out of a possible 15 canonical N-glycosites present in the protein at 83 NVS, 115 NLT, 133 NMT, 265 NET, 419 NQT, 440 NIS, 789 NLT, and 970 NDS. Only one N-glycosite at 970 was identified in male samples. Even though less abundant in microfilarial sample, Fbs1 enrichment showed this protein was N-glycosylated at 6 N-glycosites which included one at 241 NIT not found in female samples. Aligning Bm5654 with its *H. contortus* ortholog (41% identity) using BlastP^39^ shows that only the last of the 8 identified N-glycosites at 970 is shared and is NSS in *H. contortus* antigen H11.

### Adult *B. malayi* N-glycoproteins

To examine adult parasite proteins and avoid proteins that may appear in female samples due to intrauterine microfilariae, we focused on the 15 N-glycoproteins that are N-glycosylated in both female and male worms but have no identified peptides in either microfilarial Total or Fbs1 samples (Fig. 2b**, **Table 2). Because these samples were whole worm lysates, it is not possible to distinguish partial occupancy of an N-glycosite as a tissue specific N-glycosylation or the presence of two differentially glycosylated proteins in the same tissue.

**Table 2:** Adult worm N-glycoproteins.

Wormbase ID	Description	GO terms	aa length	Canonical	# of identified N-glycosites				
					All	Female		Male	
						Total	Fbs1	Total	Fbs1
Bm10329		Loc: extracellular	286	1	1		1		1
Bm10521		CC: membrane Loc: cell membrane	992	8	3		1		2
Bm10905		Loc: extracellular	165	3	2		2		1
Bm11652a	Calcium binding domain & EGF domain containing protein	MF: calcium ion binding Loc: cytoplasm	2522	29	7		5		2
Bm14109	lipid transporter protein, putative	BP: lipid transport MF: lipid transporter activity	3125	12	9	5	7		1
Bm1593	phosphodiesterase/pyrophosphatase family	MF: nucleic acid binding MF: hydrolase activity MF: metal ion binding Loc: cytoplasm	687	12	3		3		1
Bm18146	serine-type endopeptidase inhibitor activity	BP: neg reg of endopeptidase activity MF: serine-type endopeptidase inhibitor activity Loc: nucleus soluble	200	1	1		1		1
Bm3266		MF: lipid binding Loc: extracellular	971	5	2	1	2		1
Bm3610	N-acetylglucosaminyltransferase enzyme, putative	CC: Golgi membrane Loc: ER soluble	483	8	5	5	5	2	3
Bm5143	transporter, putative	BP: transmembrane transport MF: transmembrane transporter activity CC: membrane Loc: Lysosome/Vacuole Membrane	501	4	1		1		1
Bm5701	Lectin C-type domain	Loc: extracellular	674	11	4	3	4		1
Bm7870	Thrombospondin type 1 domain containing protein	Loc: extracellular	1112	6	2		2	1	1
Bm7980	Bma-spon-1 (extracellular matrix glycoprotein) is predicted to enable serine-type endopeptidase inhibitor activity	BP: neg reg of endopeptidase activity MF: serine-type endopeptidase inhibitor activity Loc: nucleus soluble	799	6	2		2		2
Bm8085	Transthyretin-like family protein	CC: extracellular space, cell surface Loc: extracellular	139	1	1	1	1	1	
Bm8157	ShTK domain containing protein	Loc: extracellular	503	3	2		2	1	1
		BP: Biological Process							
		MF: Molecular Function							
		CC: Cellular Component							
		Loc: subcellular localization							

Bm14109 is an example of a relatively abundant protein (98 unique peptides in female Total and 47 unique peptides in male Total) with variable N-glycosylation patterns found in adult male and adult female worms but not found in microfilariae. This protein is a lipid transporter protein based on gene ontology. In previous proteomic studies, it was found in adult female extracellular vesicles ^30^, adult worm surface samples^31^, and membrane samples^31^ signifying a possible host parasite interaction. This is strengthened by a recent spatial transcriptomic study, where it is enriched in the head, a druggable host-parasite interface, as well as in the intestine where drug and vaccine candidates from “hidden antigens” restricted to the alimentary canal are present^72^. In our study, eight out of 12 canonical N-linked glycosites were identified in female adult worms at sites 131 NNT, 548 NET, 634 NFT, 1187 NRT, 1524 NAT, 1638 NLT, 1692 NLS, and 1840 NES with the site at position 1840 also found aglycosylated in female and male Total samples. Exhibiting variability in N-glycosylation between female and male worms, only one N-glycosite at position 1897 NKT was found glycosylated in male samples. The only orthologs to Bm14109 identified by NCBI protein Blast^39^ are in the Spirurina^75^ suborder of nematodes or within Clade III of parasitic nematodes^34^. Table 3 shows the taxonomy of *Brugia malayi* as listed in NCBI^75^. This protein’s occurrence at the host parasite interface and in a mostly parasitic nematode suborder indicates that this glycoprotein could have a highly specialized role.

**Table 3:** NCBI *B. malayi* taxonomy.

Superkingdom	Clade	Kingdom	Clade	Clade	Clade	Clade	Phylum	Class	Order	Suborder	Infraorder	Superfamily	Family	Genus	Species
Eukaryota	Opisthokonta	Metazoa	Eumatazoa	Bilateria	Protostomia	Ecdysozoa	Nematoda	Chromadorea	Rihabditda	Spirurina	Spiruromorpha	Filarioidea	Onchocercidae	Brugia	*Brugia malayi*

Bm10521, Bm10329, and Bm10905 are additional glycoproteins with orthologs restricted to the Spirurina suborder of nematodes. These glycoproteins are not found in our microfilarial samples but are present in adult female and male worms. In addition, transcriptomics data shows Bm10521 transcripts are restricted to adult worms and Bm10329 and Bm10905 transcripts are more prevalent in adult worms^76,77^. Bm10521 protein is predicted to be in the membrane but is otherwise uncharacterized. Our data shows that in male worms it is glycosylated at two of eight canonical N-glycosites at 479 NSS and 806 NDT, while in female worms it is only glycosylated at a single different N-glycosite at 749 NDS. Bm10329 is predicted to be an extracellular protein by DeepLoc^53^. In previous proteomics studies, it was identified in membrane enriched samples^31^ and in the spatial transcriptomics study was found to be enriched in the intestine^72^. The one canonical site is N-glycosylated at 253 NVT in both female and male worms. This site is also found aglycosylated in female worms. Lastly, Bm10905 is restricted not only to the Spirurina suborder but to the Onchocercidae family. It is predicted to be extracellular by DeepLoc^53^ and otherwise uncharacterized. Our data shows that out of a possible 3 canonical N-glycosites, it is N-glycosylated at 2 sites in female worms at 37 NTS and 93 NET and only at site 37 in male worms. While the lack of homology to characterized proteins makes it difficult to assign any function to Bm14109, Bm10521, Bm10329 and Bm10905, that undetermined status along with their predicted and observed cellular location data makes these N-glycoproteins appealing candidates for further study.

Nematode restricted examples of N-glycoproteins in Table 2 are Bm3266 and Bm8085. Bm3266 by gene ontology is predicted to bind lipids and by DeepLoc^53^ is predicted to be an extracellular protein. RNAi of its *C. elegans* ortholog, F10D11.6, showed embryonic lethal and larval lethal phenotypes^78^. Though Bm3266 has 5 canonical N-glycosites, it is N-glycosylated at both 817 NTT and 884 NAS in female worms and only at 884 in male worms. Bm8085 is also predicted to be an extracellular protein and has a transthyretin-like (TTR) family domain. Recently, proteins with this TTR domain have been described as major antigens of human filarial infections^24^. Although its ortholog in *C. elegans* has no RNAi phenotype, likely due to redundancy, it has been shown to be important in cell to cell interactions. Specifically for *C. elegans ttr-52*, it acts as a bridge molecule that mediates apoptosis^79^. Bm8085 is N-glycosylated at its single canonical N-glycosite 29 NGT in both female and male worms. Because they do not have close orthologs in the host genome, both Bm3266 and Bm8085 may be interesting candidates for further study.

Bm3610 is only found in male and female worms with five out of eight canonical N-glycosites identified in female worms at 131 NIS, 227 NMT, 272 NGS, 394 NRT and 477 NIS and 3 identified N-glycosites in male worms at 131, 227 and 477. It is a single pass transmembrane protein predicted to be in the Golgi by gene ontology while DeepLoc^53^ predicts that it is an ER protein/enzyme. Gene ontology molecular function predicts that it is part of the glycosyltransferase family 14. These are a well conserved but diverse family of beta-1,6-N-acetylglucosaminyltransferase enzymes that can convert linear to branching N-acetyllactoseaminoglycans or form crucial side chain branches in O-glycans. Previous proteomic studies do not agree with the predicted location for this enzyme and have found Bm3610 in the ES proteome^29^, body wall proteome^42^, surface proteome and membrane proteome^31^. The *C. elegans* orthologs, F30A10.4, R07B7.6, and F35H8.2, are other glycosyltransferases, with the latter being annotated as a Golgi membrane protein and N-glycosylated at three N-glycosites^21^. Notably, the N-glycosites for these and other *C. elegans* orthologs identified by BlastP^39^ have no overlap with Bm3610 N-glycosylation sites and the protein identity between these nematode orthologs is below 40%. When looking for human orthologs using BlastP^39^, we find enzymes like C2GnT3^80^, a mucin type O-glycan branching enzyme with 30% protein identity. And as with the *C. elegans* orthologs, there is no overlap with the C2GnT3 N-glycosylation sites. This points to the possibility that even though it falls within the same family of glycosyltransferases, Bm3610 may play a different role or be active on a different substrate especially if this enzyme is present on the surface as indicated by the proteomic studies.

### Nematode orthologs

**Figure 6:**
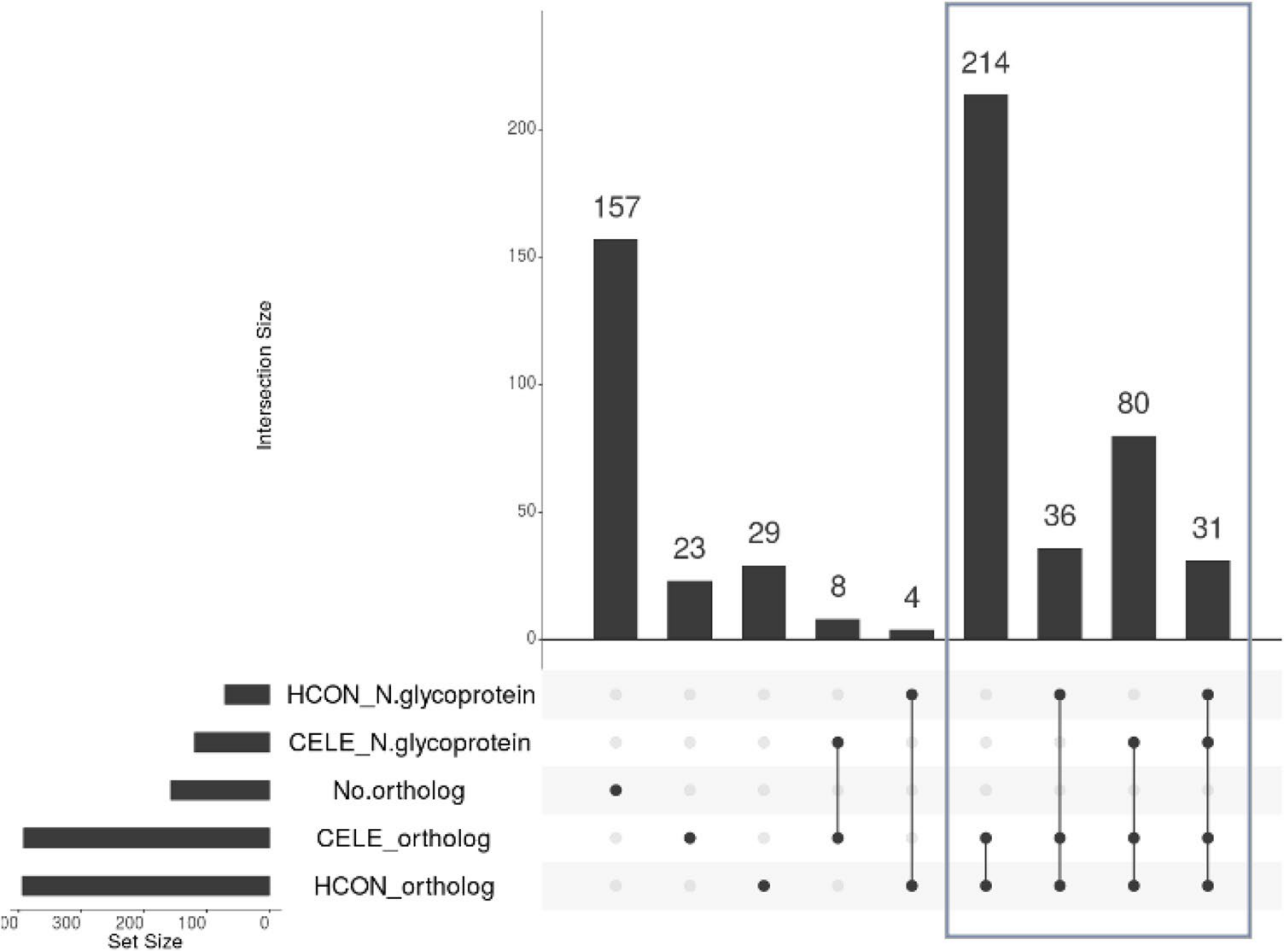
UpSetR data analysis of orthologs to *B.* *malayi* N-glycoproteins in *C. elegans* and *H.contortus: *HCON_N-glycoprotein indicates all *H. contortus* orthologs that have been identified as N-glycoproteins. HCON_ortholog indicates remaining *H. contortus* orthologs that were not identified as N-glycoproteins. CELE_N-glycoprotein indicates all *C. elegans* orthologs that have been identified as N-glycoproteins.* CELE_ortholog* indicates remaining *C. elegans* orthologs that were not identified as N-glycoproteins. No* ortholog *indicates that there were no identified* orthologs* in either *C. elegans* or *H. contortus*. The set size shown on the left indicates the number of proteins in each set. The bars that are above single dots show the number of N-glycosylated proteins unique to that* set of proteins*. The bars that are above multiple joined dots show the number of N-glycosylated proteins in common with those set of proteins. The gray box shows the *B.* *malayi* N-glycoproteins that had orthologs in both species.

Nematodes are a diverse and large group of organisms that have been organized into five clades. Both *C. elegans* and *H. contortus* belong to Clade V nematodes while *B. malayi* and other filarial nematodes are in a distinct group of Clade III nematodes^34^. We expanded on our findings by using the *C. elegans* and *H. contortus* N-glycoproteomes to search for N-glycoproteins in common and N-glycoproteins unique to *B. malayi*. We first searched for orthologs of the identified *B. malayi* N-glycoproteins in these two nematode proteomes^54^. We identified orthologs for 425 of 582 *B. malayi* N-glycoproteins leaving 157 filarial or *B. malayi* specific N-glycoproteins that have no identified ortholog in *C. elegans* or *H. contortus* (Supplemental Table S6*C. elegans* and *H. contortus* orthologs, Fig. 6). Unsurprisingly, as *C. elegans* and *H. contortus* are both Clade V nematodes and closely related to each other, 361 *B. malayi* N-glycoproteins had orthologs in both species (Fig. 6: boxed in gray). Only 31 *B. malayi* N-glycoproteins orthologs were unique to *C. elegans* (Fig. 6: 2nd & 4th bars) and 33 unique to *H. contortus* (Fig. 6: 3rd and 5th bars). We then checked if the *C. elegans* or *H. contortus* orthologs were present in their respective N-glycoproteomes^21,32,35^. While 31 *B. malayi* N-glycoproteins have both *C. elegans* and *H. contortus* orthologs that have also been identified as N-glycoproteins, 119 *B. malayi* N-glycoproteins have *C. elegans* orthologs that are also N-glycoproteins and 71 *B. malayi* N-glycoproteins have *H. contortus* orthologs that are also N-glycoproteins. This shared set of N-glycoproteins between *B. malayi*, *C. elegans* and *H. contortus* can be further explored to identify important proteins that are unique to the nematode life cycle and are unique or different from the mammalian host. We expect that some of these orthologous N-glycoproteins will have N-glycosites that are aligned or are conserved and others that will differ as found for ES-62 and H11 antigen mentioned earlier. We explored this further by using a multiple alignment^55^ of a well conserved N-glycoprotein, integrin beta, that is present in all three nematode N-glycoproteomes (Supplemental Fig. S2). Bm7611 beta integrin has seven identified N-glycosites out of a possible eight canonical N-glycosites at 54 NYT, 276 NNS, 407 NAS, 537 NES, 679 NET, 700 NDT and 728 NLT with three sites glycosylated in all three samples, one site unique to male samples and 2 sites unique to microfilarial samples. In the *C. elegans* beta integrin ortholog (72% identity), eight N-glycosites were identified which included five aligning N-glycosites at 54, 276, 407, 537, 679, and 700 as well as a different N-glycosite at 143 NVT that is not a canonical N-glycosite in *B. malayi* but is shared in the *H. contortus* sequence. A second identified site is unique to *C. elegans* at 400 NAS and is not present in the other two orthologs. The *H. contortus* integrin beta ortholog (72% identity) has two identified N-glycosites at 407 and 537. It does not have canonical N-glycosites that align with 276 or 679 but does align with the remaining identified *B. malayi* N-glycosites which might suggest that these sites are N-glycosylated at other stages of the *H. contortus* nematode life cycle. Thus, the differences observed at the aligned or conserved N-glycosites highlight the possibility for N-glycosite occupancy variation based on tissue expression or on life stages. Additionally, the 157 N-glycoproteins without orthologs are a promising set of proteins to explore as they are possibly filarial or *B. malayi* specific N-glycoproteins.

## Conclusion

Along with other nematode N-glycoproteomes, and individual *B. malayi* N-glycoproteins, this mapping of 1273 *B. malayi* N-glycosites in 582 N-glycoproteins from adult male worms, adult female worms, and microfilariae further adds to the proteomic data and to our current understanding of N-glycosylation of this filarial parasite. The N-glycosite mapping from Fbs1 enrichment of N-glycopeptides yielded highly enriched data sets by increasing both the number and proportion of identified N-glycosites while decreasing the background from N + 3 peptides without canonical N-glycosite motifs. Gene ontology and cell localization prediction showed that the N-glycoproteome was enriched for membrane and extracellular proteins. We showed that this set of N-glycoproteins can be mined in different ways. Characterization of N-glycosite occupancy of individual proteins as noted for highly glycosylated proteins listed in Table 1 and pictured for Bm7191 in Fig. 3 showed variations that could point to biological differences and needs to be explored further to determine its significance. Similarly, exploring the N-glycosylation of previously identified cuticle and immunomodulating proteins in these three host stages confirmed variation in N-glycosite occupancy present in these biologically important proteins. Investigating groups of proteins like the adult worm restricted set and how they relate to the parasite biology led to identification of parasite and nematode specific N-glycoproteins at the host interface. Those N-glycoproteins identified without host orthologs are promising therapeutic or biomarker candidates. As a follow up to this work, we plan to characterize the Fbs1 enriched peptides without PNGase F cleavage to study the intact N-glycopeptides and correlate glycan structures with specific glycosites^81^. As well as confirming our N-glycosite mapping and occupancy study, characterizing the N-glycan structures and their heterogeneity at each site for male worms, female worms, and microfilariae will yield a comprehensive understanding of the N-glycosylation of filarial parasite proteins.

## Supplementary Information

Supplementary Legends.

Supplementary Tables.

Supplementary Figures.

## Data Availability

The mass spectrometry proteomics data have been deposited to the ProteomeXchange Consortium via the PRIDE^38^ partner repository with the dataset identifier PXD039002 and 10.6019/PXD039002.
