# Supplementary material for: Defining the filarial N-glycoproteome by glycosite mapping in the human parasitic nematode Brugia malayi
Source: Sci Rep. 2023 May 16;13:7951. doi: 10.1038/s41598-023-34936-9 (PMC10187950; doi:10.1038/s41598-023-34936-9)
Supplement: Supplementary file 1 — Supplementary Legends. [file 41598_2023_34936_MOESM1_ESM.docx]

**Supplemental Information**

**Supplemental Table S1: Peptides** lists each identified peptide with sample information, Wormbase ID, sequence, start amino acid position, end amino acid position, modified amino acid, modification, presence of canonical site in the peptide, XIC (extracted ion chromatogram for the peptide) of each replicate, sum average XIC, and the standard deviation. In the last column, a * indicates a single unique peptide present in only one of the replicates of one sample and not found in other 5 samples. These peptides were not included in data analysis.

**Supplemental Table S2: Protein List** contains a compilation of the 2438 proteins that were identified from two or more unique peptides present in one or more of the six samples or from a single unique peptide independently identified in different samples. Table lists # of N-glycosites, # of unique peptides in six samples, # of replicates if single unique peptide, presence in previously published proteomes, UniProt ID, and other identifiers.

**Supplemental Table S3: N-glycosites** lists all the peptides with an identified N-glycosite. The N-glycosite table lists each N-glycosite found for each N-glycoprotein with number of N-glycosites on the protein (Glycosite multiplicity), representative peptide sequence, position of N-glycosite, presence in six samples as N-glycosite (N) or as aglycosylated site (A), and whether an aglycosylated peptide was also identified. In addition, if an N-glycosite was present only in Total samples and not in any Fbs1 sample, an asterisk is included (N*). These peptide identifications only identified in Total samples were included in our data only if that modified peptide had multiple replicates or in multiple samples.

**Supplemental Table S4: Gene enrichment** lists gene enrichment analysis of the *B. malayi* N-glycoproteins. Gene enrichment analysis was preformed using g:Profiler^48^ and the table lists each GO term and KEGG pathway identified along with associated p-values, and gene identification terms.

**Supplemental Table S5: Subcellular localization prediction by DeepLoc** contains data generated to predict subcellular localization of *B. malayi* N-glycoproteins using DeepLoc-1.0^53^.

**Supplemental Table S6: Orthologs to *B. malayi* N-glycoproteins in *C. elegans* and *H. contortus*** contains data from search for orthologs in both *C. elegans* and *H. contortus* using Parasite BioMart at Wormbase^54^ to first identify orthologs in both of *B. malayi* N-glycoproteins. The *C. elegans* published N-glycoproteins and *H. contortus* N-glycoproteins were compared to identified *B. malayi* N-glycoprotein orthologs.
