# Supplementary material for: Defining the filarial N-glycoproteome by glycosite mapping in the human parasitic nematode Brugia malayi
Source: Sci Rep. 2023 May 16;13:7951. doi: 10.1038/s41598-023-34936-9 (PMC10187950; doi:10.1038/s41598-023-34936-9)
Supplement: Supplementary file 3 — Supplementary Figures. [file 41598_2023_34936_MOESM3_ESM.docx]

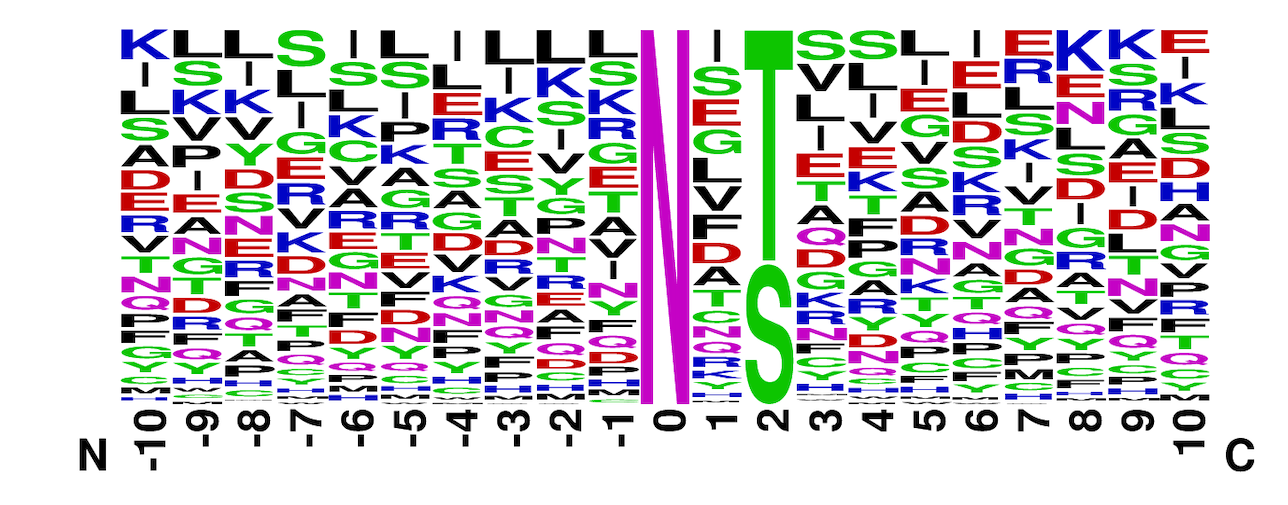


**Supplemental Figure S1: Sequence logo of B. malayi N-glycosites**: multiple sequence alignment of amino acids from -10 to +10 from 1273 N-glycosites to search for conserved motifs.


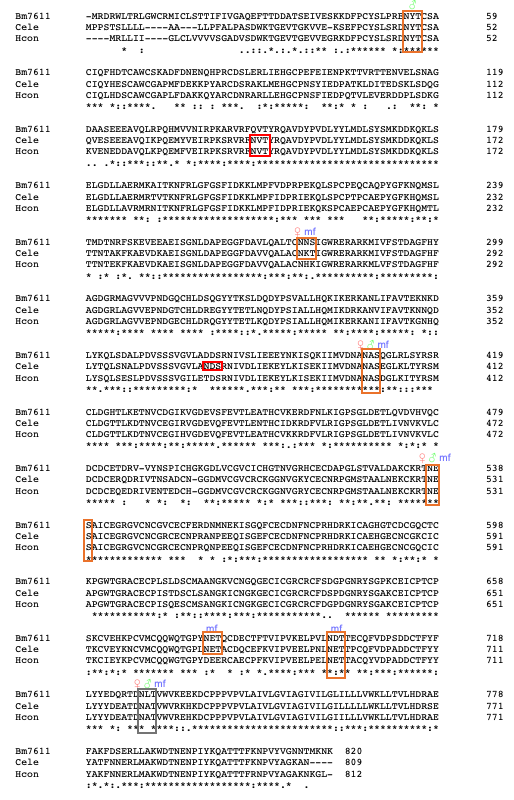


**Supplemental Figure S2: Multiple alignment of Bm7611 integrin beta with C. elegans and H. contortus orthologs**: The seven identified N-glycosites in B. malayi samples are boxed in orange if also N-glycosylated in one or more orthologs and in gray if only identified as N-glycosylated in B. malayi. N-glycosites only found in C. elegans and H. contortus orthologs are boxed in red. N-glycosites found in B. malayi female worms (♀), male worms (♂) and microfilariae (mf) are indicated above the sequence. The sequence noted as Cele is C. elegans integrin beta ZK1058.2 and the sequence noted as Hcon is H. contortus integrin beta HCON_00067860. Below the aligned sequences, a star indicates positions which have a single, fully conserved residue. A colon (:) indicates conservation between groups of strongly similar properties. A period (.) indicates conservation between groups of weakly similar properties.
